# Does Meaning-Making Mediate COVID-19 Restrictions’ Impact on Grief and Psychological Symptoms?

**DOI:** 10.1177/00302228241292388

**Published:** 2024-10-11

**Authors:** João Batista, Sara Albuquerque, Mayra Delalibera, João T. Oliveira, Alexandra Coelho

**Affiliations:** 1 CIPsi - Psychology Research Center, School of Psychology, 56059 University of Minho, Braga, Portugal; 2 HEI‐Laboratory: Digital Human‐Environment Interaction Laboratories, 70887 Lusófona University, Lisbon, Portugal; 3 37829 Faculty of Medicine of the University of Coimbra, Coimbra, Portugal; 4 AppsyCi, 56068 ISPA - University Institute, Lisboa, Portugal; 5 PsyLab, Faculty of Medicine, University of Lisbon, Lisbon, Portugal

**Keywords:** pandemic, grief, trauma, anxiety, depression, meaning-making

## Abstract

The COVID-19 pandemic imposed substantial restrictions on funeral ceremonies, profoundly affecting grief experiences. This study investigated the mediating role of meaning-making in the relationship between these restrictions and prolonged grief disorder (PGD) symptoms, anxiety, depression, and trauma. A longitudinal study involving 141 bereaved individuals was conducted, with assessments at two time points: 3–6 months (T1) and 9–12 months (T2) post-loss. Structural equation modeling revealed that the psychological impact of restrictions on death and funeral ceremonies had indirect effects on anxiety and prolonged grief symptoms through meaning-making, particularly the footing in the world subscale. These findings underscore the importance of understanding the complex interplay between pandemic-related restrictions and grief experiences, emphasizing the pivotal role of meaning-making in adapting to loss during those challenging times.

The COVID-19 pandemic imposed massive restrictions on funeral ceremonies (Wallace et al., 2020). In Portugal, as in other western countries, funeral ceremonies lacked several cultural elements, like the contact between persons, the possibility of seeing the deceased’s body, and to pay homage to the deceased person, for example, by holding ceremonies and attending religious services. These restrictions represent a novel contextual factor, altering profoundly the grief experience (Masur & Wertheimer, 2021). According to a study conducted in Portugal, mourners felt that funeral rituals were inadequate during that period (Aguiar et al., 2022). Another significant impact has been the impossibility of people to be with their loved ones at the time of death. Due to the loss of opportunities for meaningful communication, emotional connection, and physical closeness, this physical distance can be upsetting for both the dying person and their loved ones (Delor et al., 2021; Dennis et al., 2022).

Recent research has highlighted the profound impact of the COVID-19 pandemic on the grieving process. Namely, Eisma and Tamminga (2020) showed that adults who recently lost a loved one during the pandemic exhibit significantly higher levels of grief than those who experienced a loss before the pandemic. The added stressors of the pandemic, including economic instability and limited access to support services, may also increase the risk of developing or exacerbating mental health issues such as depression and anxiety. In fact, according to studies done in Portugal, mourners displayed high levels of trauma, depression, and prolonged grief symptoms during the pandemic (Alves, 2023).

The goal of the current study is to better understand how COVID-19-related restrictions affected mourning by examining meaning-making as a potential mediator of this relationship. Although other mediators were studied in grief research (e.g., social support and rumination), we considered that the degree of integration of the loss during the lockdown could be a more meaningful mediator of the impact of the restrictions on prolonged grief, anxiety, depression, and trauma symptoms.

Meaning-making can be broadly defined as a “mental representation of possible relationships among things, events, and relationships. Thus, meaning connects things” (Baumeister, 1991, p. 15). According to Park (2010), meaning-making entails four main components: 1) global meaning, which is the person’s view of himself, others, and the world; 2) situational meaning, the evaluation made after an event, such as threat, loss, challenge, etc.; 3) the meaning-making processes used to reduce the discrepancy between the global meaning and the meaning of an event, that is, all the efforts made by the individual to understand that event, such as the possibility of having to create new meanings; and, finally, 4) the product of the meaning-making process, which refers to the final meaning.

Despite the theoretical complexity of the meaning-making construct and its measurement (see Park & George, 2013 for details), a pivotal idea is that it can be central in the recovery of stressful or traumatic events by allowing the integration of the event that violated the person’s global meanings (Park & George, 2013). One approach to grief that has shown promising results is the meaning reconstruction model (Gillies & Neimeyer, 2006). This model focuses on the integration of loss into the person’s global meaning system, either through the assimilation of the loss or the accommodation of the existing meanings (Holland et al., 2010). This model led to the development of several measures of meaning-making, including the Integration of Stressful Life Experiences Scale (Holland et al., 2010) used on this study.

Research with this model suggests that meaning-making processes are relevant to the grief process. Rozalski et al. (2017), for example, showed that the ability to find meaning in loss fully mediated adaptation to the violent death of a significant other (e.g., suicide, homicide, accidents). Similarly, Milman et al. (2018) discovered that the ability to make sense of loss mediated the impact of a number of risk factors (e.g., violent death loss, low social support, attachment insecurity) on the intensity of prolonged grief symptomatology.

The COVID-19 pandemic was a sudden and global phenomenon with widespread impact on people’s lives. In this sense, several studies investigated the role that meaning-making could have in the adaptation to the pandemic. For instance, Milman et al. (2020) argued that core belief violations and decreased meaning-making of the pandemic would be relevant in the impact of COVID-19 stressors in mental health. This assertion was tested and confirmed with a large sample (*N* = 2380), in which both variables were found to mediate the impact of COVID-19 direct and indirect stressors on anxiety and depression. Breen et al. (2022) also showed that meaning-making partially mediated the relationship between the pandemic risk factors for grief with functional impairment and dysfunctional grief symptoms. These findings seem to consistently indicate that meaning-making processes may be significant in the adaptation to the COVID-19 pandemic, especially when a highly stressful event, such as a loss, occurred during that period of time.

## The Present Study

Given the impact that COVID-19 had on mental health and on grieving in particular (e.g., Eisma et al., 2020), it is paramount to identify the processes that influenced such negative effects, both to mitigate them and to prevent future situations derived from similar phenomena. This study addresses the question of the process of meaning-making and explains the relationship between the lockdown restrictions on death and funeral ceremonies and the evolution of grief-associated symptoms in the Portuguese population. In this sense, this study intends to confirm the pivotal role that meaning-making seems to have had in the adaptation to the pandemic, addressing the restrictions that everyone lived under and not only the risk factors of COVID-19.

This is a quantitative, longitudinal, prospective, and observational study with two moments of assessment during the mourning period: T1: 3–6 months after the death of the loved one; T2: 9–12 months after the death of the loved one. The hypotheses for this study were the following: 1) Meaning-making will mediate the impact of restrictions on symptoms of prolonged grief disorder; 2) Meaning-making will mediate the impact of restrictions on symptoms of anxiety; 3) Meaning-making will mediate the impact of restrictions on symptoms of depression; 4) Meaning-making will mediate the impact of restrictions on symptoms of trauma.

## Method

### Participants

Participating in the study were 141 bereaved people who answered a questionnaire online or by telephone in two moments. The following requirements had to be met in order to be eligible: (1) having lost a close relative during the pandemic by COVID-19, regardless of the reason for death; (2) being at least eighteen years old; (3) possessing Portuguese literacy; and (4) having at least three months since the death to avoid the crisis period.

### Procedure

This study is part of a larger study that received approval from the Ethics Committee of the two regional health administrations. Data collection occurred from October 2020 to June 2021. Multiple strategies were employed to ensure participant recruitment, including developing a website with participation instructions and disseminating the information through personal connections, social media, and various hospitals nationwide. The questionnaires could be completed online (83.6%), through a link to the participant to answer, or by telephone (16.4%), to ensure that people with no internet access or lacking technological proficiency could participate. The informed consent form guaranteed the confidentiality, anonymity, and voluntary nature of all data collected.

Dissemination of the study was interrupted during festive seasons (e.g., Christmas), as these are periods that can be emotionally activating. Additionally, sociodemographic questions were collected at the end so that participants finished with less emotional charge. Participants were provided the contact information of local grief consultations, the Psychological Support Line, and an emergency health line upon request.

### Measures and Instruments

#### Sociodemographic Information Questionnaire

A questionnaire constructed by the researchers assessed the characteristics of the participant (e.g., age, gender, marital status) and of the deceased (age and gender), as well as the circumstances of the loss (e.g., time post-loss, cause, and place of death).

#### Restrictions and Psychological Impact of the Restrictions Questionnaire

This questionnaire was developed by the research team based on literature and discussion with peers and health professional specialists and it was used at T1. It included 10 items referring to post-death, including the moment of death (e.g., “be present at the moment of death,” “see and touch the body”) and mourning rituals (e.g., “perform the funeral mass,” “visit the cemetery”). For each item, participants were instructed to assess the extent to which they were restricted (“In comparison to what you would have wanted, for each of the following, indicate to what extent you felt constrained by the restrictions”) and the intensity of its psychological impact (“To what extent did the following restrictions impact you?”) in a five-point Likert scale, from 1 (*none*) to 5 (*extremely*). The mean scores for each subscale were calculated and examined separately, with higher values indicating the greater extent and psychological impact of pandemic restrictions on bereavement experience. Internal consistency was tested by computing Cronbach’s alpha for the subscales Identification of post-death restrictions (α = .87), and Psychological impact of post-death restrictions (α = .90).

#### Impact of Event Scale 6

The IES-6 (Thoresen et al., 2010; adapted by Lopes, 2013) measured symptoms of Post Traumatic Stress Disorder (PTSD) at T1 and T2 (Thoresen et al., 2010; Lopes, 2013). It is a brief continuous Likert scale with six items, with five options each, *from not at all* (0) to *extremely* (4). Scores range from 0 to 24; higher scores reflect higher levels of symptoms of PTSD. The Portuguese version of this scale revealed good internal consistency, α = .84 (Lopes, 2013). The cutoff of the IES- 6 is 12.5. Cronbach’s α values for this study were .85 at T1 and .88 at T2.

#### Prolonged Grief Disorder Scale-13

The PG-13 (Prigerson et al., 2007; adapted by Delalibera et al., 2011) assessed Prolonged Grief Disorder (PGD) symptoms at T2. This scale has 13 items; 11 items assess cognitive, behavioral, and emotional symptoms (Delalibera et al., 2011; Prigerson et al., 2007). Five items are scored on a Likert scale from 1 *(not at all*) to 5 (*several times a day*), and six are rated on an intensity scale from 1 (*not at all*) to 5 (*overwhelmingly*). The duration and impairment items are dichotomous (*yes/no*). The score of the scale is calculated from the sum of the 11 items on a Likert scale, with a total score ranging from 11 to 55. Higher values represent increasing levels of PGD symptoms severity. The Portuguese validation presented excellent internal consistency, α = .93 (Delalibera et al., 2011). In order to score (diagnose) Prolonged Grief Disorder (PGD) the following must be met: criteria A - event criterion - that is having experienced the death of a close person; criteria B - separation distress - the respondent must experience PG-13 questions #1 or 2 at least daily; criteria C - duration criterion - the symptoms of separation distress must be elevated at least 6 months after the loss. that is, pg-13 question #3 must be answered as “yes”; criteria D - cognitive, emotional, and behavioral symptom - the respondent must experience 5 of the PG-13 questions #4–12 at least “once a day” or “quite a bit”; criteria E - impairment criterion - the respondent must have significant impairment in social, occupational, or other important areas of functioning (e.g., domestic responsibilities). that is, PG-13 question #13 must be answered as “yes”. In the current sample, the PG-13 presented good internal consistency with α = .95.

#### Brief Symptom Inventory

The BSI (Derogatis & Melisaratos, 1983; adapted by Canavarro, 1999) was used to measure depression and anxiety at T2 using the 12 items of the scale for those dimensions (Canavarro, 1999; Derogatis & Melisaratos, 1983). This inventory has a Likert scale ranging from 0 (*not at all*) to 4 (*extremely*). The Portuguese version of the BSI showed good psychometric qualities, with the internal consistency values for each subscale presenting Cronbach’s α values between .62 and .80 (Canavarro, 1999). In the present sample, α was .91 for the dimension Depression and .92 for the dimension Anxiety at T2.

#### Integration of Stressful Life Experiences Scale

The ISLES (Holland et al., 2010; adapted by Albuquerque et al., 2024) was used at T2 to assess meaning-making. It has 16 items measured on a Likert scale, ranging from 1 (*strongly disagree*) to 5 (*strongly agree*) (Albuquerque et al., 2024; Holland et al., 2010). The scale includes two subscales: 1) comprehensibility, the extent to which a person has been able to make sense of a stressful event such as a loss; and 2) footing in the world, the extent to which the world makes sense following a stressful event. Higher scores in the ISLES indicate a greater ability to integrate the loss, and lower values may show some maladjustment to the stressful experience (Holland et al., 2010). The Portuguese version of this scale (Albuquerque et al., 2024) revealed good internal consistency for both Comprehensibility (α = .84) and Footing in the world (α = .94) subscales. Cronbach’s α value for this study was .87 for Comprehensibility and .93 for footing in the world at T2.

### Data Preparation and Statistical Analysis

There was no missing data as, for the online questionnaire, we used mandatory fields to require responses for key questions and for the telephone survey, we monitored responses in real-time to correct any gaps immediately. Visual assessments using pairwise scatterplots and correlation coefficients explored the relationships between the independent variables (IVs), restrictions and the psychological impact of restrictions at death and funeral ceremonies, the mediator (ISLES total scale and subscales), and the dependent variables (DVs), prolonged grief, anxiety, depression, and trauma symptoms. Structural equation models (SEMs) were created for the VIs and VDs that showed a significant association with the mediators, using the *lavaan* package (Rosseel, 2012). Standardized coefficients and parameter estimates, complemented by bootstrap confidence intervals, were derived for the SEM models. Mediation analyses were conducted to thoroughly assess the indirect effects. The mediation function was applied with 10.000 bootstrap samples. All statistical analyses were conducted using R software (version 4.2.2; R Core Team, 2022).

## Results

Most of the 141 bereaved who took part in the study were women (87.9%), married (61%), had more than 12 years of education (71.6%), and were working full time (81.6%). The average age of participants was 41.18 years (SD = 13.91). Most of the bereaved lost their father/mother (50.4%), and 41.8% of the deceased had an oncological disease (41.8%), and 65.2% of the bereaved lived with the deceased. The characteristics of the sample and the deceased and circumstances of loss are shown in Tables 1 and 2. At T1, 4% of participants were at risk for prolonged grief disorder (PGD), and 80.1% had trauma symptoms. At T2, the prevalence of PGD increased to 8.5%, while 70.9% still showed trauma symptoms.

**Table 1.** Demographic Characteristics of the Sample.

Variable	Category	*N* (%)	M (SD)
Gender	Male	17 (12.1)	
	Female	124 (87.9)	
Age	—	—	41.18 (13.9)
Marital status	Single	32 (22.7)	
	Married/consensual union	86 (61)	
	Divorced	12 (8.5)	
	Widowed	11 (7.8)	
Education	Less than 12 years	8 (5.7)	
	12 years	32 (22.7)	
	More than 12 years	101 (71.6)	
Occupation	Unemployed	6 (4.3)	
	Student	7 (5.0)	
	Full-time job	115 (81.6)	
	Retired	5 (3.5)	
	Sick leave	4 (2.8)	
	Inactive	4 (2.8)	
Relationship with the deceased	Spouse/partner	11 (7.8)	
	Father/mother	71 (50.4)	
	Grandfather/grandmother	31 (22.0)	
	Another relative	21 (14.8)	
	Friend	7 (5.0)	
Time since loss	T1	—	5.44 (3)
	T2	—	11.13 (3.6)

**Table 2.** Demographic Characteristics of the Deceased and Circumstances of Loss.

		*n* (%)
Deceased		
Age of the deceased *M* (DP)		76 (13.8)
Gender of deceased	Female	76 (53.9)
	Male	65 (46.1)
Cause of death	Cancer	59 (41.8)
	Organ failure	37 (26.2)
	Neurodegenerative	5 (3.5)
	Other	21 (15)
Place of death	Hospital	79 (56)
	Home	29 (20.5)
	Nursing home	14 (10)
	Palliative care	16 (11.4)
	Other	3 (2.1)

### Preliminary Analysis

Two-way Pearson correlations showed a significant association between the psychological impact of restrictions at death and funeral ceremonies with prolonged grief (r = .21, *p* < .005) and anxiety (r = .24, *p* < .005) symptoms at T2, but not with any other DV. No associations were found between the identification of restrictions and the DVs. A significant correlation was found between the psychological impact of restrictions at the death and funeral ceremonies and the footing in the world subscale of the ISLES (r = .22, *p* < .01). Finally, the footing in the world subscale showed significant associations both with prolonged grief (r = .79, *p* < .005) and anxiety (r = .70, *p* < .001) at T2. In sum, one IV (psychological impact of restrictions at death and funeral ceremonies), one mediator (footing in the world subscale) and two DVs (prolonged grief and anxiety symptoms) presented significant associations and were included in the SEMs.

### Structural Equation Models (SEM) and Mediation Analysis

#### First SEM Analysis (Anxiety) and Mediation Results

**Figure 1.**
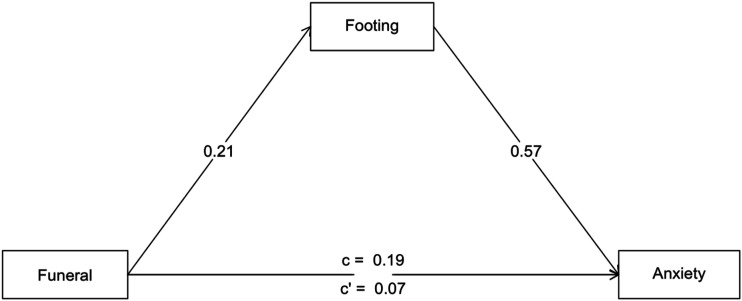
Path diagram illustrating the mediation effect of footing on the relationship between funeral restrictions and anxiety.

In the first SEM (Figure 1), the psychological impact of restrictions at death and funeral ceremonies had a significant positive direct effect on footing in the world (β = .21, 95% CI [.06, .36], *p* = .007) and anxiety had a significant positive direct effect on footing in the world (β = .57, 95% CI [.45, .68], *p* < .001). These results suggest that both the psychological impact of restrictions and anxiety are associated with decreased footing in the world. Additionally, the SEM indicated that the psychological impact of restrictions had a significant indirect effect on anxiety through footing in the world (β = .12, 95% CI [.03, .22], *p* = .010), suggesting that footing in the world mediates the relationship between the psychological impact of restrictions and anxiety.

The mediation analysis for anxiety (Table 3) confirmed the mediating role of footing in the world between the psychological impact of restrictions at death and funeral ceremonies and anxiety. The total effect of the psychological impact of restrictions on anxiety was significant (c = .19, 95% CI [.05, .32], *p* = .005), whereas the direct effect was not significant (c' = .07, 95% CI [−.02, .17], *p* = .16). The indirect effect of the psychological impact on anxiety through footing in the world was significant (β = .12, 95% CI [.03, .21], *p* < .05), indicating that footing in the world accounts for a significant portion (63%) of the total effect of the psychological impact on anxiety.

**Table 3.** Parameter Estimates and 95% Confidence Intervals for SEM Model 1 (Anxiety).

Parameter	Estimate (β)	95% CI Lower	95% CI Upper	*p*-Value	Standardized Estimate
Direct effects					
Footing ∼ funeral	.21	.06	.36	.007	.22
Anxiety ∼ footing	.57	.45	.68	<.001	.68
Anxiety ∼ funeral (c’ path)	.07	−.02	.17	.147	.09
Indirect effects					
Funeral → anxiety (via footing)	.12	.03	.22	.010	.15
Total effects					
Funeral → anxiety	.19	.05	.32	.006	.24

*Note.* Confidence intervals are bootstrapped.

#### Second SEM Analysis (Prolonged Grief) and Mediation Results

**Figure 2.**
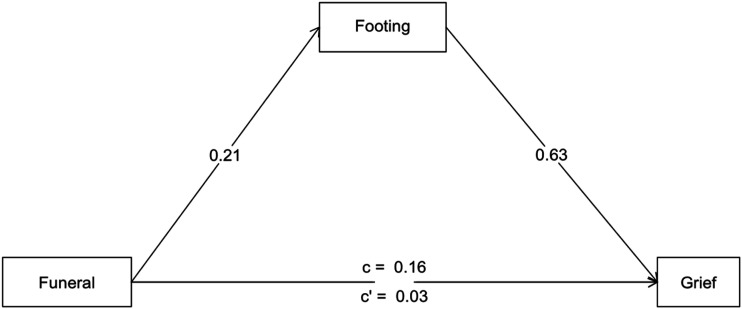
Path diagram illustrating the mediation effect of footing on the relationship between funeral restrictions and prolonged grief.

The second SEM (Figure 2) demonstrated a significant positive direct effect of the psychological impact of restrictions at death and funeral ceremonies on footing in the world (β = .21, 95% CI [.05, .36], *p* = .009) and a significant positive direct effect of footing in the world on grief symptoms (β = .63, 95% CI [.53, .73], *p* < .001). These findings suggest that the psychological impact of restrictions is associated with decreased footing in the world, which in turn is associated with grief symptoms. The SEM also indicated a significant indirect effect of the psychological impact of restrictions on grief through footing in the world (β = .13, 95% CI [.03, .23], *p* = .009), suggesting that footing in the world mediates this relationship.

The mediation analysis for grief symptoms (Table 4) further confirmed the mediating role of footing in the world between the psychological impact of restrictions and grief. The total effect of the psychological impact on grief was significant (c = .16, 95% CI [.03, .28], *p* = .014), while the direct effect was not significant (c' = .02, 95% CI [−.05, .12], *p* = .53). The indirect effect of the psychological impact on grief through footing in the world was significant (β = .14, 95% CI [.03, .23], *p* < .05), indicating that footing in the world explains a substantial portion of the relationship between the psychological impact of restrictions and grief.

**Table 4.** Parameter Estimates and 95% Confidence Intervals for SEM Model 1 (Grief).

Parameter	Estimate (β)	95% CI Lower	95% CI Upper	*p*-Value	Standardized Estimate
Direct effects					
Footing ∼ funeral	.21	.05	.36	.009	.22
Grief ∼ footing	.63	.53	.73	<.001	.78
Grief ∼ funeral (c’ path)	.03	−.05	.12	.484	.04
Indirect effects					
Funeral → grief (via footing)	.13	.03	.23	.009	.17
Total effects					
Funeral → grief	.16	.03	.28	.010	.21

*Note*: Confidence intervals are bootstrapped.

## Discussion

This study aimed to understand whether the process of meaning-making explains the relationship between the lockdown restrictions on death and funeral ceremonies and the evolution of grief-associated symptoms in the Portuguese population.

We explored if meaning-making was a mediator of the effects of COVID-19 lockdown restrictions in death and funeral ceremonies on symptoms of trauma, prolonged grief, anxiety, and depression. The mediation models showed that meaning-making was a partial mediator of anxiety and prolonged grief symptoms, more specifically the footing in the world subscale.

As aforementioned, meaning-making measured as an adaptation to a stressful event has been consistently proven to be a relevant variable in the grief process (e.g., Rozalski et al., 2017). However, most of these studies were done with specific populations (e.g., widowers or parents who lost their offspring) and not with a community sample. Thus, the present study seems to add to the evidence of the central role of meaning-making in the aftermath of a loss with a heterogeneous sample and to align with results of other studies done during the pandemic (Milman et al., 2020). Moreover, it also points out the importance of fostering some understanding of an uncommon event, like COVID-19. The significant association with footing in the world subscale seems to reflect the need to find an orientation within the context of a pandemic and a loss; within unforeseen and potential life events, coping involves the reconstruction of meaning, of a viable assumptive world (Jannoff-Bulman, 1992).

In addition, the restrictions on death and funeral ceremonies were associated with meaning-making. This seems to point out the relevance of these ceremonies in the grief process; rituals of mourning are relevant to the grief process, as they promote acceptance of the reality of loss, enable emotional expression and social support, and foster adaptive meanings of continuing bonds with the deceased (Albuquerque et al., 2021; Mitima-Verloop et al., 2021). According to recent research (Burrell & Selman, 2020; Chen, 2022), the inability to do these rituals as a result of pandemic limitations has had a significant effect on grief experiences.

Also, these restrictions likely impact the bereaved individuals’ ability to be present and engage in meaningful interactions with their loved ones during their final moments and the time of death. This is particularly important within the context of our study, given that the majority of the participants experienced the loss of their loved ones due to prolonged illnesses, particularly cancer, with most deaths occurring within hospital settings. Given the context of the study, compounded by pandemic restrictions, the hospital setting as the place of death likely had significant implications for the bereaved, which aligns with findings in existing literature (Dennis et al., 2022; Delor et al., 2021). Particularly, as shown in our study, such limitations can hinder the grieving process by interfering with the formation of adaptive coping mechanisms, such as the process of meaning-making.

In short, our study highlights the role of meaning-making in navigating grief amid COVID-19 restrictions, underscoring the significance of death and funeral ceremonies in the grieving process. Understanding these dynamics is crucial for supporting individuals facing loss during those unprecedented times as well as in future mass death and pandemic health concerns.

While this study revealed that meaning-making played a significant role as a mediator in mitigating symptoms of anxiety and prolonged grief derived from the restrictions, its impact did not extend similarly to symptoms of trauma and depression. The divergence in the mediation effect suggests that the mechanisms through which meaning-making operates might vary depending on the specific manifestation of grief-associated symptoms. For instance, anxiety and prolonged grief symptoms may be more closely intertwined with individuals’ efforts to find meaning and make sense of their loss, thus rendering meaning-making a salient mediator in alleviating these aspects of grief. On the other hand, symptoms of trauma and depression may involve complex psychological processes that extend beyond the scope of meaning-making alone, necessitating a multifaceted approach to address these facets of grief-associated symptoms. Further research is important to investigate deeper into the underlying mechanisms driving these distinctions.

### Limitations

Regarding the study’s limitations, having a larger sample size would provide a more robust basis for drawing conclusions and generalizing findings. Also, while the study employs a longitudinal design with assessments at two time points, the findings may not capture the long-term trajectory of grief (after the assessed 12-month time frame). Moreover, the mediator was only assessed at T2, instead of between the measurement of independent and dependent variables. This is a limitation of cross-sectional studies, such as the present one, that preclude the examination of causal relations between the variables. Although we assumed, based on the literature, that meaning-making would be a mediator of anxiety, depression, trauma, and prolonged grief, the association of these variables with the restrictions may be more complex.

Understanding how mediators operate at different time points can inform interventions and support strategies for bereaved individuals at early and later stages of grief. Nonetheless, the findings of this study are in line with the research done in the wake of the pandemic, showing that meaning-making was a relevant factor in the adaptation of the population to loss in that period of time.

## Data Availability

The data that support the findings of this study are available from the corresponding author upon reasonable request.*
